# Increased Expression of Cannabinoid CB_1_ Receptors in Achilles Tendinosis

**DOI:** 10.1371/journal.pone.0024731

**Published:** 2011-09-08

**Authors:** Emmelie Björklund, Sture Forsgren, Håkan Alfredson, Christopher J. Fowler

**Affiliations:** 1 Pharmacology Unit, Department of Pharmacology and Clinical Neuroscience, Umeå University, Umeå, Sweden; 2 Section for Anatomy, Department of Integrative Medical Biology, Umeå University, Umeå, Sweden; 3 Sports Medicine Unit, Department of Surgical and Perioperative Sciences, Umeå University, Umeå, Sweden; Alcon Research, Ltd., United States of America

## Abstract

**Background:**

The endogenous cannabinoid system is involved in the control of pain. However, little is known as to the integrity of the cannabinoid system in human pain syndromes. Here we investigate the expression of the cannabinoid receptor 1 (CB_1_) in human Achilles tendons from healthy volunteers and from patients with Achilles tendinosis.

**Methodology:**

Cannabinoid CB_1_ receptor immunoreactivity (CB_1_IR) was evaluated in formalin-fixed biopsies from individuals suffering from painful Achilles tendinosis in comparison with healthy human Achilles tendons.

**Principal Findings:**

CB_1_IR was seen as a granular pattern in the tenocytes. CB_1_IR was also observed in the blood vessel wall and in the perineurium of the nerve. Quantification of the immunoreactivity in tenocytes showed an increase of CB_1_ receptor expression in tendinosis tissue compared to control tissue.

**Conclusion:**

Expression of cannabinoid receptor 1 is increased in human Achilles tendinosis suggesting that the cannabinoid system may be dysregulated in this disorder.

## Introduction

It has been known for many years that cannabinoids are effective for the relief of a variety of types of pain [1], and activation of cannabinoid (CB) receptors by compounds such as nabilone have been shown to have clinical utility in a variety of pain states, including cancer pain, neuropathic pain, and fibromyalgia [2]–[4]. Data from studies on experimental animals indicate that the antinociceptive effects of cannabinoids are not only centrally mediated, but that spinal and peripheral CB receptors are involved [5].

The antinociceptive effects of exogenous CB receptor agonists raise the possibility that the endocannabinoid system is dysfunctional in pain states. There is evidence of this in animal models of pain [6]–[10], but little is known about the situation in human pain. To our knowledge, the only data so far reported are findings of an increased plasma concentration of the endocannabinoid anandamide in patients with complex regional pain syndrome compared to age- and sex-matched controls [11], a negative correlation between CB_1_ receptor expression in pancreatic nerves and strong pain symptoms in patients with pancreatic cancer [12], and a positive correlation of suburothelial CB_1_-immunoreactive nerve fibers with the pain score in painful bladder syndrome [13]. There is thus a need for more data on the status of the endocannabinoid system in human pain states.

The Achilles tendon is the strongest tendon in the body and the understanding of pain in this tendon is of utmost importance. When there is chronic pain and impaired function in the Achilles tendon, the condition is referred to as tendinopathy. Clinically, the tendon thickens and becomes tender. Biopsies show changes in the appearance of the tenocytes, hypercellularity and neovascularization and eventually degenerative features [14]–[19]. When there are structural changes, the condition is commonly called tendinosis [20].

Achilles tendinosis most often occurs in the mid-portion of the tendon. Imaging with ultrasonography or magnetic resonance imaging (MRI) can be used to verify tendon abnormalities in the painful area [18], [21]. The pain in chronic Achilles tendinosis remains an enigma. However, injections and mini-surgery to areas of neovascularization outside the tendon have been shown to reduce the pain seen in chronic Achilles tendinosis [22], [23]. Although several molecular candidates have been identified and proposed as mediators of the pain in Achilles tendinosis [24], information on the cannabinoid system is lacking. In consequence, we have investigated the expression of CB_1_ receptors in human Achilles tendons and whether there is a change in expression in Achilles tendinosis.

## Methods

### Ethics Statement

The Committee of Ethics at the Faculty of Medicine, Umeå University and the Regional Ethical Review Board in Umeå approved the project (04-157 M). Participants gave verbal consent to participate in the research after reading an explanatory statement and receiving a verbal summary of the project. The Review Board approved the verbal consent procedure. When approval from patients had been obtained, specimens were collected and taken care of for the research and documentation was made. This form of approval and documentation is in accordance with paragraph 16 and 17 of the Ethical review act in Sweden. All procedures followed the principles of the Declaration of Helsinki.

### Individuals

This investigation included tissue samples from 24 individuals: 11 males and 13 females (mean age: 48 years, range 21–70 years; male mean age: 44 years, range 28–70 years; female mean age: 52 years, range 21–68 years). The samples were from a group of patients suffering from Achilles tendinosis and from healthy controls. The Achilles tendinosis group (*n = 17*) were suffering from chronic painful mid-portion Achilles tendinosis. This group consisted of 8 males and 9 females (mean age: 51, range 28–70 years; male mean age: 45 years, range 28–70; female mean age: 57 years, range 47–68 years). Tendinosis was verified by ultrasonography. The control group consisted of individuals (*n = 7*) with no history of pain symptoms from their Achilles tendons (mean age: 41 years, range 21–47 years; 3 males, mean age 41 years, range 39–46 years; 4 females, mean age: 41, range 21–47 years). Ultrasonography showed normal tendons. All patients were otherwise healthy and non-smokers.

### Sampling, fixation and sectioning

In the tendinosis group, biopsies were taken during surgical treatment. Under local anaesthesia (4–5 ml pilocaine hydrochloride, 10 mg/ml; AstraZeneca, Södertälje, Sweden), tendon tissue (macroscopically abnormal) was taken from the ventral part of the Achilles tendon through a longitudinal incision lateral to the tendon mid-portion. The biopsies contained tendon tissue proper, and to varying degrees, outer parts of the tendon (paratendinous connective tissue). The tissue samples were approximately 2 mm wide and 1–5 mm long and were taken from different depths of the tendon. From the control group, samples of the same size were collected from the dorsal part of the tendon using a longitudinal plain incision under local anaesthesia. The dorsal part of the tendon was chosen for ethical and practical reasons.

Directly after the surgical procedures, the samples were fixed overnight at 4°C in a solution of 4% formaldehyde in 0.1 M phosphate buffer, pH 7.0, followed by thorough washing in Tyrode's solution containing 10% sucrose, at 4°C overnight. The samples were then mounted on thin cardboard in OCT embedding medium (Miles Laboratories, Naperville, IL,USA) and frozen at −80°C until sectioning.

For the immunofluorescence and morphologic investigations, series of 7 µm thick sections from both groups of samples were cut using a cryostat (Leica Microsystem CM3050S, Heidelberg, Germany). The sections were mounted on slides, pre-coated with crome-alun gelatine, and the sections were then dried and thereafter processed for immunohistochemistry. Staining with hematoxylin-eosin was carried out to delineate tissue morphology. Reference tissues (human colonic and rat dorsal root ganglion tissue), available at the laboratory, were examined in parallel. They had been fixed and further processed in the same way as the tendon samples.

### Immunofluorescence

Sections were initially treated with acid potassium permanganate for 2 min to enhance the visualization of specific immunofluorescence reaction sites [25]. The sections were then rinsed in 0.01 M phosphate-buffered saline (PBS) pH 7.4 containing 0.1% sodium azide as preservative, for 3×5 min. After these procedures, incubation for 20 min in a 1% solution of detergent Triton X-100 (Kebo Lab, Stockholm, Sweden) in 0.01 M PBS, and rinsing for 3×5 min in PBS were performed. The sections were then incubated in 5% normal swine serum in PBS supplemented with 0.1% bovine serum albumin (BSA) for 15 min in a humid environment in room temperature. Thereafter followed incubation with the primary antibody, diluted in PBS with BSA. The incubation was performed in a humid environment and proceeded for 60 min at 37°C. The sections were then washed in PBS for 3×5 min prior to another incubation in normal swine serum for 15 min in a humid environment in room temperature, followed by incubation with the secondary antibody. This antibody corresponded to tetramethylrhodamine isothiocyanate (TRITC)- conjugated swine antirabbit igG (DakoCytomation, Glostrup, Denmark). The secondary antibody was diluted in PBS with BSA (1∶40). Incubation proceeded for 30 min at 37°C in a humid environment. A Zeiss Axioskop II microscope equipped with an Olympus DP70 digital camera was used for examination of the sections.

For semiquantitative determination of the intensity of CB_1_ receptor immunoreactivity in the control and Achilles tendinosis tendons, two of the researchers (EB, SF), who were blinded to the clinical data for the patients, independently scored the CB_1_ expression in the sections under the fluorescence microscope. The tenocytes were scored for immunoreactive intensity (0–3 where 0 is absent and 3 is high), and the average value was taken. This method of scoring has previously been used in other published studies by co-author Forsgren [26], [27], including studies using the immunofluorescence technique on tendon tissue [28]. For the CB_1_ receptor immunoreactivity scores returned by the two investigators, an intraclass coefficient analysis using a two-way mixed effects model for the 24 scores gave a Cronbach's alpha reliability coefficient of 0.79, suggesting that the inter-rater reliability is acceptable.

### Antibodies and control stainings

The antibodies used were against CB_1_ and PGP9.5. Both are rabbit antibodies. The CB_1_ antibody (rabbit polyclonal; ab23703; Abcam, Cambridge, UK) had been raised against the C-terminal amino acid 461–472 of human CB_1_. Concentrations used were 1∶20–1∶100. For the experiments comparing immunoreactivity for controls and tendinosis patients, a dilution of 1∶50 was used. The antibody to PGP9.5 (rabbit polyclonal; code 7863-0504; Biogenesis, Poole, UK), was used at a dilution of 1∶100, and had been raised against native brain PGP9.5.

For control purposes, sections were processed with PBS/BSA instead of primary antibodies. To confirm further the specificity, the primary CB_1_ antibody was pre-absorbed overnight at 4°C with its immunogenic peptide (20–100 µg/ml; ab50542; Abcam, Cambridge, UK) prior to incubation on the sections. The PGP9.5 antibody has been used in numerous studies in the laboratory [29], [30] and in numerous studies by others for the purpose of demarcating nerve fibres.

### Statistics

The two-tailed Mann-Whitney test was undertaken using the Statistical package built into the GraphPad Prism 5 computer programme for the Macintosh (GraphPad Software Inc., San Diego, CA, USA). The intraclass coefficient analysis was conducted using IBM SPSS Statistics 19 for the Macintosh (IBM Inc., Somers, NY, USA).

## Results

### CB_1_ receptor immunoreactivity (CB_1_IR) in the normal Achilles tendon and in reference tissue

**Figure 1:**
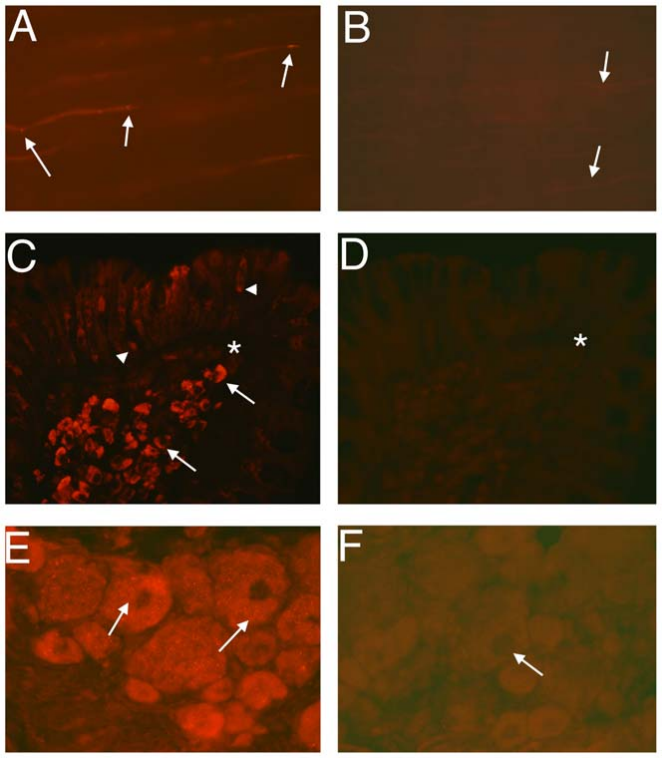
Immunofluorescence for CB_1_ in normal Achilles tendon and in reference tissue. Panels show sections processed for CB_1_ (**a,c,e**) and with CB_1_ antibody preabsorbed with CB_1_ antigen (**b,d,f**). Immunoreactions are shown in the elongated tenocytes in (**a**) but not in (**b**). Arrows indicate tenocytes. Immunoreactions are also seen in cells of the mucosa and submucosa (arrows) and the epithelial layer (arrowheads) of human colon in (**c**) but not in (**d**), and in cell bodies of a rat dorsal root ganglion in (**e**) but not in (**f**) (arrows). Asterisks in similar region in (**c**) and (**d**). Original magnification ×40.

Figure 1 shows the CB_1_IR for normal Achilles tendons, in which the tendon cells (tenocytes) show the characteristic elongated appearance [18], [19], [31] and, for comparative purposes, that for human colon and the rat dorsal root ganglion. For all three cases, CB_1_IR was seen. For the tendons, CB_1_IR was observed for the tenocytes (Fig. 1A) and in blood vessel walls (not shown). The reactions in the tenocytes showed a punctuate appearance along the length of the cells (Fig. 1A). Preabsorption with the corresponding peptide eliminated the immunostainings (Fig. 1B), as did control stainings when the primary antibody was omitted (data not shown). As positive controls, CB_1_IR was investigated in the human colon and the dorsal root ganglia. For the human colon, distinct specific reactions were seen for cells of the mucosa and submucosa and in cells in the epithelial layer (Fig. 1C), in agreement with the literature [32]. Distinct specific reactions were also seen for neuronal perikarya of the dorsal root ganglion. The reactions occurred as granular intracellular reactions (Fig. 1E). The presence of intracellular CB_1_IR can also be seen in the literature [33]. For both human colon and the rat dorsal root ganglia, preabsorption with the corresponding peptide eliminated the immunostainings (Fig. 1D and F).

### CB_1_IR in Achilles tendinosis

**Figure 2:**
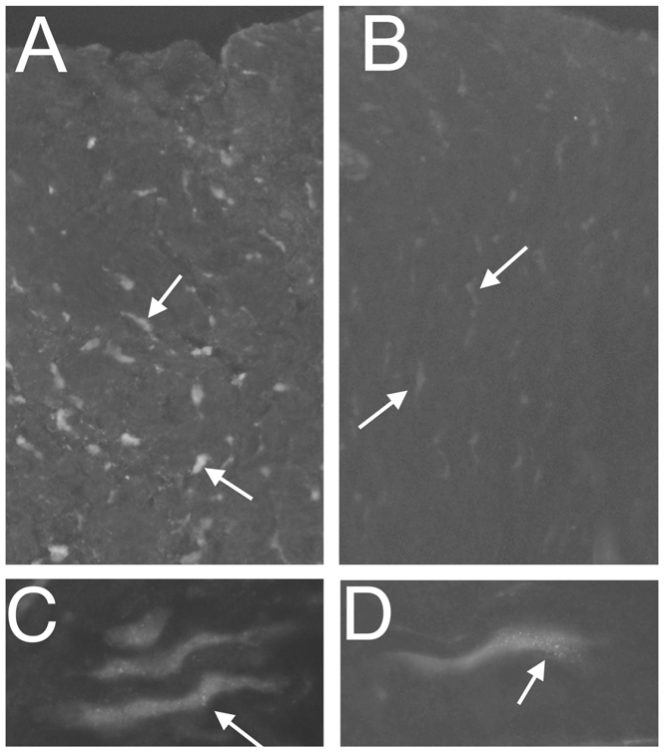
Immunofluorescence for CB_1_ in Achilles tendinosis. Panels show sections of Achilles tendinosis tendons processed for demonstration of CB_1_ (**a,c,d**) and of CB_1_ after preabsorption with the immunogenic peptide (**b**). Numerous tenocytes are seen in low magnification in (**a**) and (**b**) (arrows at tenocytes). They show specific immunoreactions in (**a**) but not in (**b**). In panel **c** and **d** in which the tenocytes are shown in high magnification, punctuate immunoreactions in tenocytes are shown (arrows). Original magnification ×20 (**a,b**), ×63 (**c,d**).

The CB_1_IR pattern in tenocytes of samples obtained from patients with Achilles tendinosis is shown in low magnification in (Fig. 2A–B), where Fig. 2B shows that preincubation with the antigen prevents all the immunoreactive staining. At a higher magnification, a granular pattern of immunoreactivity was seen for the tenocytes. That was especially the situation for rounded/swollen tenocytes and tenocytes with a wavy appearance. These abnormally-formed tenocytes are a characteristic of Achilles tendinosis [16], [19]. The tenocyte reactions appeared overall to be the strongest in the tendinosis specimens.

### CB_1_IR in blood vessel walls and nerve structures

**Figure 3:**
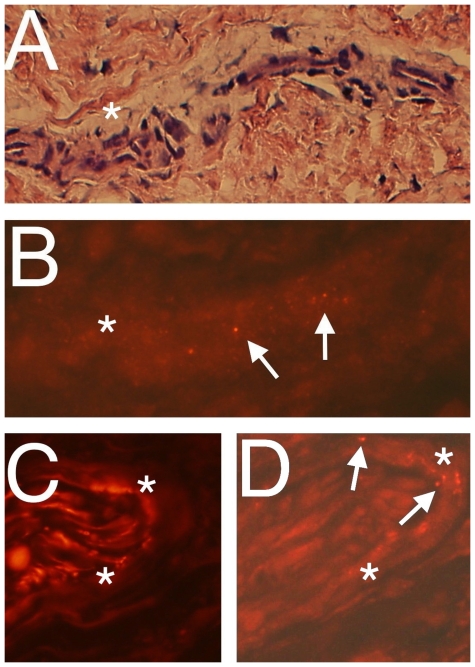
Immunofluorescence for CB_1_ in blood vessel and nerve fasicle. Panels show sections of Achilles tendinosis tissue showing a small blood vessel (**a,b**) and a part of a nerve fascicle (**c,d**) stained with htx-eosin (**a**), processed for CB_1_IR (**b,d**), and for PGP9.5 (**c**). Immunoreactions (arrows) are seen in the blood vessel wall (**b**) and in the perineurium of the nerve fascicle (arrows) (d). Original magnification ×20 (**a**), ×40 (**c,d**), ×63 (**b**). Asterisks in similar region in (**a**) and (**b**) and in the perineurium in (**c**) and (**d**).

CB_1_IR was also observed in blood vessel walls and in the perineurium of nerves in the samples, especially in the tendinosis samples. For the blood vessel walls (confirmed by htx-eosin staining Fig. 3A), CB_1_IR was in the form of fine pointed reactions (Fig. 3B). They were seen for small vessels but not for large vessels. Preabsorption with the immunising peptide confirmed specificity of the antibody (data not shown).

Nerve fascicles were demarcated via showing a distinct PGP9.5 immunoreaction (Fig. 3C). CB_1_IR was detected in the perineurium of the nerve fascicles. No such reactions were, on the other hand, detected within the interior of the nerve fascicles (Fig. 3D). The nerve related immunoreactions were abolished following preabsorption with the antigen peptide (data not shown).

### Semiquantitative comparison of tenocyte CB_1_IR in controls and patients with Achilles tendinosis

**Figure 4:**
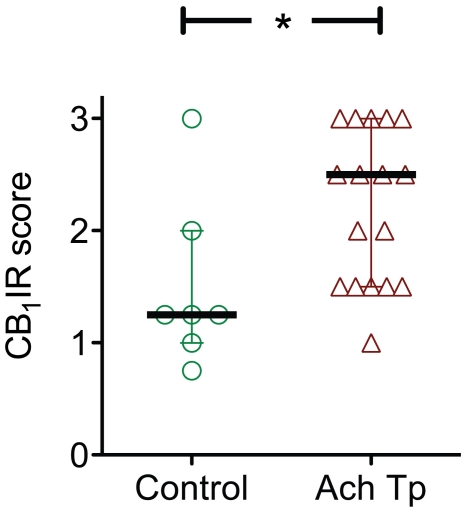
CB_1_IR scores for biopsy samples from controls and patients with Achilles tendinopathy. Shown is a box and whiskers plot of CB_1_ immunoreactivity in pain-free Achilles tendons (controls, n = 7) vs. tendons from patients with Achilles tendinopathy (n = 17). *p = <0.05, two-tailed Mann-Whitney U test. Values are mean of the scoring made by the two investigators.

A semiquantitative analysis of the reaction intensities for the tenocytes was performed. CB_1_ was scored in tenocytes from a total of 24 patients either suffering from chronic Achilles tendinosis or having clinically pain-free Achilles tendons. Difference in CB_1_ expression between groups was statistically significant (p<0.05, Mann-Whitney U test) (Fig. 4). It should be noted that the control patient with the highest CB_1_IR was presumably asymptomatic at the time of biopsy, but that the tendon showed pathology consistent with Achilles tendinopathy, thus questioning whether this sample is a true control. Exclusion of this sample from the data set increased the level of significance to p<0.005.

## Discussion

In this study we demonstrate, for the first time, that CB_1_IR is detectable within the tenocytes of the human Achilles tendon. Immunoreactions were also seen in the walls of small blood vessels walls and in the perineurium of nerves. On the other hand, whilst CB_1_IR was detectable in the neuronal perikarya of the rat spinal ganglion, CB_1_IR was not detectable in the nerves of tendon tissue.

The antibody used for these studies, raised against the C-terminal amino acid 461–472 sequence of human CB_1_, has been found previously to produce the appropriate staining in brain samples, but not to stain samples from CB_1_ knockout mice [34], [35]. The antibody has been used, by us and others, to investigate CB_1_ receptor expression pattern in human bladder [36] and fallopian tube [37], and in colorectal and prostate cancer biopsies [34], [35]. We noted that immunoreactivity was present for non-neuronal cells and that it was not limited to the cell plasma membranes, but that intracellular reactions were also seen. Similarly we noted in the present study that the CB_1_IR was detectable intracellularly in the neuronal cell perikarya and in the cells of the mucosa/submucosa and the epithelial cells of the colon. Although at first sight this may seem strange for a G-protein coupled receptor, it is known that the majority of the endogenous CB_1_ receptors do not reach the cell surface [38], and several authors have reported the presence of intracellularly located CB_1_ receptors in immunohistochemical and western blot experiments [35], [38]–[41]. Furthermore, there is evidence in neuroblastoma cells that at least some of the intracellular CB_1_ receptors are functionally active and couple to the extracellular signal-regulated kinase-signalling pathway [38]. A recent study shows that anandamide, an endogenous CB_1_ receptor agonist, can activate intracellular CB_1_ receptors and provide evidence that these are functional [42].

The function of the CB_1_ receptors in the tenocytes can at present only be a matter of speculation, but the present data add to the literature indicating that these cells are not simply inert cells but express receptors for a variety of signalling molecules that presumably affect their function [19], [43]–[46]. Interestingly, tenocytes in the human Achilles tendon have been found to show expression of transporters or enzymes favouring that these cells can produce nerve signal signal substances, such as acetylcholine [44], glutamate [46] and catecholamines [19]. They were also found to show expression of mRNA for substance P [43]. They show in addition expression of receptors for neurotransmitters [19], [43]–[44]. These features were clearly obvious in tendinosis tendons and most clearly so for tenocytes showing abnormal appearances, whilst such features were very vague or not detected at all in normally appearing tenocytes. This means that tenocytes can develop “neuronal-like” characteristics in terms of expressions of nerve signal substances and expressions of receptors for these. It is also a well-known fact that there are no nerve fibres within the Achilles tendon tissue as such, the existing innervations being present in the outer part of the tendon (the peritendinous tissue) and to a very small extent in the connective tissue septa [30]. These findings may suggest that biochemical mediators, such as nerve signal substances, produced in the tendon tissue, can be involved in the aetiology and pathogenesis of chronic tendon pain in tendinosis [47]. A “biochemical model” of this type, involving other locally produced signal substances, has also previously been considered [48]. This means that the pain is not only structural in origin. To what extent such a model is related to what we see here, i.e. an up-regulation of CB_1_IR for tenocytes in tendinosis tendons, the CB_1_ immunoreactions being especially obvious for tenocytes with an abnormal morphology, remains to be further explained. In any case, our observations show that the cannabinoid receptor system in the Achilles tendon is mainly related to the tendon cells and not being confined to the nerve fibres.

In the present study, the robust and reliable semiquantitative determination method used, also found that there was a higher CB_1_ receptor expression level in the tenocytes of patients with Achilles tendinosis than in the control group, suggesting that the cannabinoid system may be dysregulated in this disorder. It is possible that the increased expression may be an adaptive consequence to a loss of local endocannabinoid signalling, and it would clearly be of interest to investigate local endocannabinoid levels in microdialysis studies from this patient group.

An alternative explanation is that the increased CB_1_ receptor expression is due to changes in its cellular regulation produced by the local environment in the disorder. There is evidence in the literature that inflammatory processes can affect expression of CB_1_ receptors in some cases, but not others: Izzo et al. [49] for example demonstrated that croton oil-induced intestinal inflammation resulted in a three-fold increase in CB_1_ receptor expression in the jejunum. In contrast, cystitis produced by intravesical administration of acrolein did not affect bladder CB_1_ investigation at the time points chosen [50]. CB_1_ and CB_2_ receptors can be regulated by both pro- and anti-inflammatory cytokines [51], and a good example of this is the ability of interleukin-4 (IL-4) to increase CB_1_ receptor expression in lymphocytes by a Stat-6 mediated pathway [52], [53]. Tenocytes in primary culture express interleukin-4 (IL-4) receptors [45]. However, inflammation presumably occurs only at the beginning of tendinosis, and there are no signs of inflammation at the time when the biopsy samples used here were taken, so that if the increased expression found in the present study emanates from the early inflammation, it would have to be a very long-lasting effect, or due to the residual presence of a factor long after resolution of the inflammation. This does not, of course, rule out the possibility that other factors, i.e. not associated with inflammation, are involved in the regulation of CB_1_ receptor expression in the tendons. Experiments in both patient tissue and animal models of Achilles tendinopathy [54] are clearly needed to explore further the mechanisms behind the increased CB_1_ receptor expression in Achilles tendinosis.

In conclusion, the present study has demonstrated the expression of CB_1_ receptors in Achilles tenocytes, thereby adding to the growing list of non-neuronal cell types that express these receptors, and that their expression is increased in tendinosis. These data underline the need for further studies on endocannabinoid signalling in human pain conditions.
